# Evolving Digital Ecological Networks

**DOI:** 10.1371/journal.pcbi.1002928

**Published:** 2013-03-07

**Authors:** Miguel A. Fortuna, Luis Zaman, Aaron P. Wagner, Charles Ofria

**Affiliations:** 1 Integrative Ecology Group, Estación Biológica de Doñana (EDB-CSIC), Sevilla, Spain; 2 Department of Ecology and Evolutionary Biology, Princeton University, Princeton, New Jersey, United States of America; 3 BEACON Center for the Study of Evolution in Action, Michigan State University, East Lansing, Michigan, United States of America; University of Toronto, Canada

## Abstract

“*It is hard to realize that the living world as we know it is just one among many possibilities*” [1]. **Evolving digital ecological networks** are webs of interacting, self-replicating, and evolving computer programs (i.e., digital organisms) that experience the same major ecological interactions as biological organisms (e.g., competition, predation, parasitism, and mutualism). Despite being computational, these programs evolve quickly in an open-ended way, and starting from only one or two ancestral organisms, the formation of ecological networks can be observed in real-time by tracking interactions between the constantly evolving organism phenotypes. These phenotypes may be defined by combinations of logical computations (hereafter tasks) that digital organisms perform and by expressed behaviors that have evolved. The types and outcomes of interactions between phenotypes are determined by task overlap for logic-defined phenotypes and by responses to encounters in the case of behavioral phenotypes. Biologists use these evolving networks to study active and fundamental topics within evolutionary ecology (e.g., the extent to which the architecture of multispecies networks shape coevolutionary outcomes, and the processes involved).

This is a “Topic Page” article for *PLOS Computational Biology*.

## Overview

“*So far, we have been able to study only one evolving system […] If we want to discover generalizations about evolving systems, we will have to look at artificial ones*” [2].

**Figure 1:**
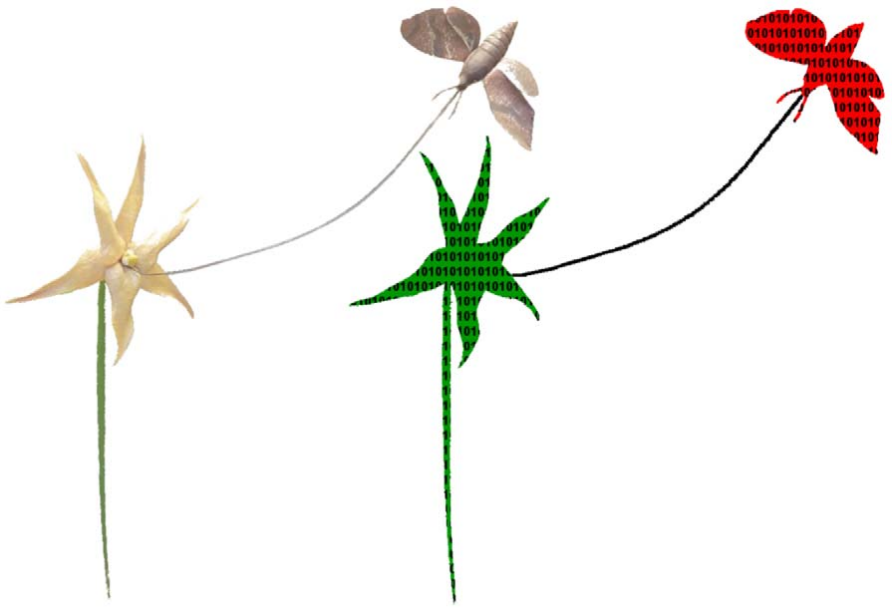
Pairwise coevolution. When Darwin received an orchid (Angraecum sesquipedale) from Madagascar whose nectary was one and a half feet long, he surmised that there must be a pollinator moth with a proboscis long enough to reach the nectar at the end of the spur [32]. In its attempt to get the nectar, the moth would have pollen rubbed onto its head, and the next orchid visited would then be pollinated. In 1903, such a moth was discovered: Xanthopan morgani. This was a remarkable example of an evolutionary prediction. However, because species coevolve within large networks of multispecies ecological interactions, this example of pairwise coevolution is more the exception than the rule.

In nature, species do not evolve in isolation but in large networks of interacting species (see Figure 1). One of the main goals in evolutionary ecology is to disentangle the evolutionary mechanisms that shape and are shaped by patterns of interaction between species [3]–[5]. A particularly important question concerns how coevolution, the reciprocal evolutionary change in local populations of interacting species driven by natural selection [6], is shaped by the architecture of food webs, plant–animal mutualistic networks, and hostparasite communities. The concept of diffuse coevolution, where adaptation is in response to a suite of biotic interactions [7], was the first step towards a framework unifying relevant theories in community ecology and coevolution. Understanding how individual interactions within networks influence coevolution, and conversely how coevolution influences the overall structure of networks, requires an appreciation for how pairwise interactions change due to their broader community contexts as well as how this community context shapes selective pressures [8], [9]. Accordingly, research is now focusing on how reciprocal selection influences and is embedded within the structure of multispecies interactive webs, not only on particular species in isolation [4].

Coevolution in a community context can be addressed theoretically via mathematical modeling and simulation [10], [11], by looking at ancient footprints of evolutionary history via ecological patterns that persist and are observable today [12], [13], and by performing laboratory experiments with microorganisms [14]. In spite of the long time scales involved and the substantial effort that is necessary to isolate and quantify samples, the latter approach of testing biological evolution in the lab has been successful over the last two decades [15]. However, studying the evolution of interspecific interactions, which involves dealing with more complex webs of multiple interacting species, has proven to be a much more difficult challenge. A meta-analysis of hostphage interaction networks, carried out by Weitz and his team [16], found a striking statistical structure to the patterns of infection and resistance across a wide variety of environments and methods from which the hosts and phage were obtained. However, the ecological mechanisms and evolutionary processes responsible have yet to be unraveled.

Digital ecological networks enable the direct, comprehensive, and real time observation of evolving ecological interactions between antagonistic and/or mutualistic digital organisms that are difficult to study in nature. Research using self-replicating computer programs can help us understand how coevolution shapes the emergence and diversification of coevolving species interaction networks and, in turn, how changes in the overall structure of the web (e.g., through extinction of taxa or the introduction of invasive species) affect the evolution of a given species. Studying the evolution of species interaction networks in these artificial evolving systems also contributes to the development of the field, while overcoming limitations evolutionary biologists may face. For example, laboratory studies have shown that historical contingency can enable or impede the outcome of the interactions between bacteria and phage, depending on the order in which mutations occur: the phage often, but not always, evolves the ability to infect a novel host [17]. Therefore, in order to obtain statistical power for predicting such outcomes of the coevolutionary process, experiments require a high level of replication. This stochastic nature of the evolutionary process was exemplified by Stephen Jay Gould's inquiry (“What would happen if the tape of the history of life were rewound and replayed?”) [18]. Because of their ease in scalability and replication, evolving digital ecological networks open the door to experiments that incorporate this approach of *replaying the tape of life*. Such experiments allow researchers to quantify the role of historical contingency and repeatability in network evolution, enabling predictions about the architecture and dynamics of large networks of interacting species.

The inclusion of ecological interactions in digital systems enables new research avenues: investigations using self-replicating computer programs complement laboratory efforts by broadening the breadth of viable experiments focused on the emergence and diversification of coevolving interactions in complex communities. This cross-disciplinary research program provides fertile grounds for new collaborations between computer scientists and evolutionary biologists.

## History

### Coreworld

The field of digital life was inspired by the rampant computer viruses of the 1980s. These viruses were self-replicating computer programs that spread from one computer to another, but they did not evolve. Steen Rasmussen was the first to include the possibility of mutation in self-replicating computer programs by extending the once-popular Core War game, where programs competed in a digital battle ground for the computer's resources [19]. Although Rasmussen observed some interesting evolution, mutations in this early genetic programing language produced many unstable organisms, thus prohibiting scientific experiments. Just one year later, Thomas S. Ray developed an alternative system, Tierra, and performed the first successful experiments with evolving populations of self-replicating computer programs [20].

### Tierra

Thomas S. Ray created a genetic language similar to earlier digital systems, but added several key features that made it more suitable for evolution in his artificial life system, Tierra. Primarily, he prevented instructions from writing beyond the *privately* allocated memory space, thus limiting the potential for organisms writing over others [20]. The only selective pressure in Tierra was for rapid self-replication. Over the course of evolution, this pressure lead to shorter and shorter genomes, reducing the time spent copying instructions during replication. Some individuals even started executing the replication code in other organisms, allowing those cheaters, which were originally referred to as parasites in Ray's work, to further shrink their genetic programs. This form of cheating was the first evolved ecological interaction between organisms in artificial life software. Ray's cheaters pre-dated the formal study of evolving ecological interactions using Tierra-like digital evolution platforms by 20 years.

### Avida

In 1993, Christoph Adami, Charles Ofria, and C. Titus Brown created the artificial life platform Avida [21] (available at http://avida.devosoft.org/download/) at the California Institute of Technology. They added the ability for digital organisms to obtain bonus CPU cycles for performing computational tasks, like adding two numbers together. In Avida, researchers can define the available tasks and set the consequences for organisms upon successful calculation [21]. When organisms are rewarded with additional CPU cycles, their replication rate increases. Since Avida was designed specifically as a scientific tool, it allows users to collect a comprehensive suite of data about evolving populations. Due to its flexibility and data tracking abilities, Avida has become the most widely used digital system for studying evolution. The Devolab (http://devolab.msu.edu/) at the BEACON Center currently continues development of Avida.

## Implementation

### Digital Organisms

**Figure 2:**
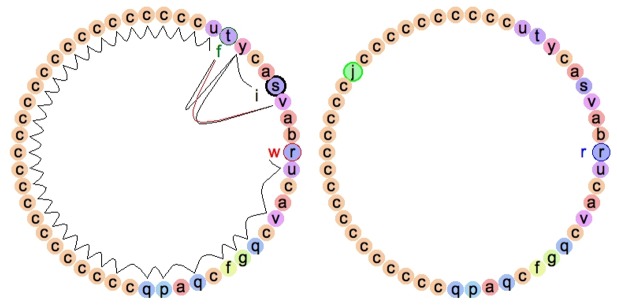
Self-replication of a digital organism. The circular genome of a digital organism, on the left, consists of a set of instructions (represented here as letters). Some of these instructions are involved in the copy process and others in completing computational tasks. The experimenter determines the probability of mutations. Copy mutations occur when an instruction is copied incorrectly, and is instead replaced by a random instruction in the forming offspring's genome (as can be seen in the offspring, on the right). Other types of mutations, such as insertions and deletions are also implemented. All three of the parent's hardware pointers are represented: the instruction pointer (indicated by an *i*), the write-head pointer (indicated by a *w*), and the flow pointer (indicated by an *f*). Arcs inside the circular genome represent the execution flow, showing most of the CPU cycles being used during the copying process. After genome replication is complete, the parent organism divides off its offspring, which must now fend for itself within the Avida world. The last snapshot of an animation representing the self-replication process of a digital organism, Video S1, is shown. It was generated using Avida-ED, which is available under the terms of the GNU Lesser General Public License at http://avida-ed.msu.edu/.

Digital organisms in Avida are self-replicating computer programs with a genome composed of assembly-like instructions. The genetic programing language in Avida contains instructions for manipulating values in registers and stacks as well as for control flow and mathematical operations. Each digital organism contains virtual hardware on which its genome is executed. To reproduce, digital organisms must copy their genome instruction by instruction (see Figure 2) into a new region of memory through a potentially noisy channel that may lead to errors (i.e., mutations). While most mutations are detrimental, mutants will occasionally have higher fitness than their parents, thereby providing the basis for natural selection with all of the necessary components for Darwinian evolution. Digital organisms can acquire random binary numbers from the environment and are able to manipulate them using their genetic instructions, including the logic instruction NAND. With only this instruction, digital organisms can compute any other task by stringing together various operations because NAND is a universal logic function [22]. If the output of processing random numbers from the environment corresponds to the result of a particular logic task, then that task is incorporated into the set of tasks the organism performs, which, in turn, defines part of its phenotype.

### Digital Interactions

**Figure 3:**
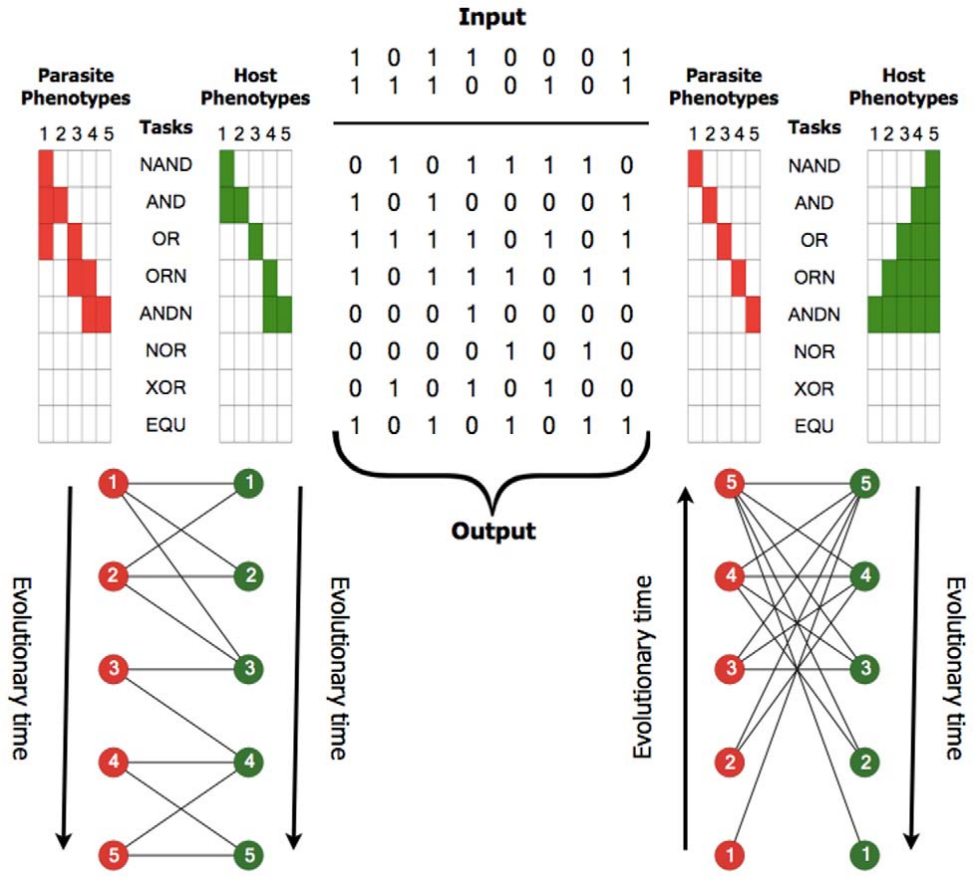
Logical computations, i.e., tasks, partially define phenotypes, and phenotypic matching leads to ecological interactions. Digital organisms process binary numbers taken from the environment using the instructions that constitute their genomes. When the output of processing those numbers equals the result of applying a logic function, the digital organism is said to have performed that task. The combination of tasks performed by a digital organism partially defines its phenotype. The center of the figure depicts the output of applying eight logical operators (tasks) on the two input numbers above. On the left and right, five hypothetical host (green) and parasite (red) phenotypes are represented as columns (on the top) and as circles (below). On the top, each column depicts a phenotype and each row represents a task. Tasks performed by each phenotype are filled. In the lower part, the interaction networks between hosts and parasites are illustrated, which result from phenotypic matching: a parasite infects a host (indicated by a line) if it performs at least one task that is also performed by the host. Inset numbers indicate the identity of phenotypes represented on the top. Arrows represent the temporal direction of the coevolutionary process: from the earliest phenotype to the most recent one. The order of tasks (from top to bottom) indicates the time needed for a digital organism to perform that task over the course of the evolutionary trajectory. Depending on the pattern of tasks performed by the digital organisms, a modular (left) or nested (right) interaction network can emerge.

Interactions between digital organisms occur through phenotypic matching, which, in the case of task-based phenotypes, results from the performance of overlapping logic functions (see Figure 3). Different mechanisms for mapping phenotypic matching to interactions can be implemented, depending on the antagonistic or mutualistic nature of the interaction.

#### Host–parasite interactions

In hostparasite interactions, the parasite organisms benefits at the expense of the host organisms. Parasites in Avida are implemented just like other self-replicating digital organisms, but they live inside hosts and execute parasitic threads using CPU cycles stolen from their hosts [23]. Because parasites impose a cost (lost CPU cycles) on hosts, there is selection for resistance, and when resistance starts to spread in a population, there is selective pressure for parasites to infect those new resistant hosts. Infection occurs when both the parasite and host perform at least one overlapping task. Thus a host is resistant to a particular parasite if they do not share any tasks (see Figure 3). This mechanism of infection mimics the inverse-gene-for-gene model [24], in which infection only occurs if a host susceptibility gene (the presence of a logic task) is matched by a parasite virulence gene (a parasite performing the same task). Additional infection mechanisms, such as the matching allele and gene-for-gene models [25], can also be implemented.

In traditional infection genetic models, host resistance and pathogen infectivity have associated costs. These costs are an important part of theory about why defense genes do not always fix rapidly within populations [26]. Costs are also present in digital host–parasite interactions: performing more or more complex tasks implies larger genomes and hence slower reproduction. Tasks may also allow organisms access to resources present in the abiotic environment, and the environment can be carefully manipulated to control the relative costs or benefits of resistance.

**Figure 4:**
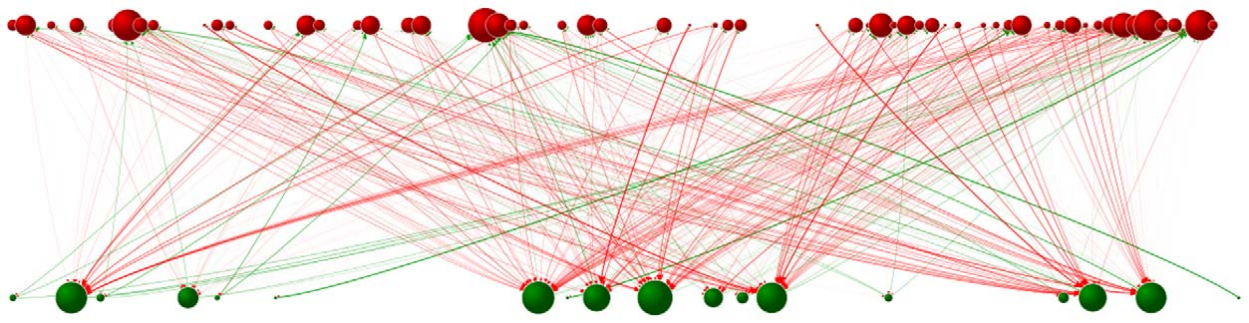
Evolving host–parasite webs. Starting from a host phenotype (green node) and a parasite phenotype (red node), a complex network of interactions (arrows) between hosts and parasites emerges out of the coevolutionary process. Nodes representing new host and parasite phenotypes appear and disappear over evolutionary time. The abundance of individuals expressing each phenotype changes continuously (indicated by node size) altering interaction patterns, and thus influencing subsequent coevolutionary dynamics. Interactions between a host phenotype and a parasite phenotype are depicted as arrows pointing in opposite directions: the thickness of red arrows indicates the fraction of infections that a particular parasite is responsible for inflicting on the indicated host phenotype, while the thickness of the green arrows indicates the fraction of all of the hosts a particular parasite phenotype infects that is accounted for by the indicated host phenotype. Often asymmetry between the thicknesses of arrow-pairs leads to red arrows dominating the picture. At these times, most parasite phenotypes are infecting only a small fraction of hosts expressing a given phenotype. Instead, the majority of those hosts are being infected by parasites with other phenotypes. This is a single snapshot of Video S2, which depicts the evolutionary dynamics of a host–parasite community. It was generated using Pajek, which is available under the GNU General Public License at http://pajek.imfm.si/doku.php.

By keeping track of task-based phenotypes as well as tracking information about successful infections in the community, researchers are able to perfectly reconstruct the interaction networks of digital coevolving hosts and parasites (see Figure 4). The structure of these networks is a result of the interplay between ecological processes, mainly host abundance, and coevolutionary dynamics, which lead to changes in host specificity. [27]

#### Mutualistic interactions

Interactions in which both species obtain mutual benefit, such as those between flowering plants and pollinators, and birds and fleshy fruits, can be implemented in evolving digital experiments by following the same task matching approach used for host–parasite interactions, but using free-living organisms instead of parasitic threads. For example, one way to set up a plant-pollinator type of interaction is to use an environment containing two mutually exclusive resources: one designated for “plant” organisms and one for “pollinator” organisms. Similar to parasites attempting infection, if tasks overlap between a pollinator and a plant it visits, pollination is successful and both organisms obtain extra CPU cycles. Thus, these digital organisms obtain mutual benefit when they perform at least one common task, and more common tasks lead to larger mutual benefits. While this is one specific way to enable mutualistic interactions, many others are possible in Avida. Interactions that begin as parasitic may even evolve to be mutualistic under the right conditions. In most cases, coevolution will result in concurrent interactions between multiple phenotypes. Thus, observed networks of mutualistic interactions can inform our understanding about the outcomes and processes of coevolution in complex communities [28].

#### Predator–prey interactions

While hostparasite and mutualistic interactions are determined by task-based phenotypes, predatorprey interactions are determined by behavior. Predators are digital organisms that have evolved from ancestral prey phenotypes to locate, attack, and consume organisms. When a predator executes an attack instruction (acquired through mutation), it kills a neighboring organism. When predators kill prey, they gain resources required for reproduction (e.g., CPU cycles) proportional to the level accumulated by the consumed prey. Selection favors behavioral strategies in prey that enable them to avoid being eaten. At the same time, selection favors predators with behavioral strategies that improve their food finding and prey attacking abilities. The resulting diversity in the continuously evolving behavioral phenotypes creates dynamic predator–prey interaction networks in which selective forces are constantly changing as a consequence of the emergence of new, and loss of old, behaviors. Because predators and prey move around in and use information about their environment, these experiments are typically carried out using spatially structured populations. On the other hand, host–parasite and mutualistic coevolution are often done in well-mixed environments, though the choice of the environment is at the discretion of the experimenter.

## Research Directions

Understanding how biodiversity is organized in natural ecosystems requires going beyond the study of pairs of interacting species. Using digital organisms, one can find generalities about the evolutionary and ecological processes shaping the web of interactions among species, as well as the coevolutionary processes embedded within these networks. By tracing the evolution of digital communities and their ecological networks, researchers obtain perfect fossil records of how the number and patterns of links among interacting phenotypes evolved.

The stability–diversity debate [29] is a long-standing debate about whether more diverse ecological networks are also more stable. Until recently, this debate has focused on one component of biodiversity: species diversity. However, newer research has begun dealing with another component of biodiversity: diversity in species interactions. Mathematical models show that a mixture of antagonistic and mutualistic interactions can stabilize population dynamics and that the loss of one interaction type may critically destabilize ecosystems [30]. Studies with digital organisms can shed light on this debate from an empirical perspective because the types of interactions included can be manipulated and the stability of the resulting evolving digital ecological network can be measured.

Equally addressable using evolving digital ecological networks are many of the open questions concerning the coevolution of ecological interactions in multispecies communities. For example, do coevolutionary dynamics change as communities become richer? Is there any limit to their richness? Is the evolution of interactions between multispecies networks historically contingent Why do some ecological scenarios lead to predictable network structures and others do not? [31] Do genetic constraints play a large role in the evolution of ecological networks? These are only a few of many open questions concerning the coevolution of ecological interactions in multispecies communities.

These and many related questions require researchers to look across the evolutionary history of ecological network formation. For natural systems, those data are very difficult to collect. With digital organisms, watching both the coevolutionary process and ecological network formation is possible in real time. Data on the abundance of interacting phenotypes are recorded without error; hence, the evolutionary implications of ecological processes can be explored in-depth.

The study of self-replicating and evolving computer programs offers a tantalizing glimpse into the evolution of interactions among organisms that do not share any ancestry with the biochemical life of Earth. This comes with potential caveats in translating predictions of evolving digital networks to biological ones because mechanistic details differ substantially between interacting digital organisms and interacting biological organisms. Nevertheless, these digital networks contain the necessary components for ongoing coevolutionary dynamics in large webs of interacting organisms. In spite of the differences between biological and digital evolution, the study of evolving digital ecological networks can lead to a more predictive understanding of natural dynamics. Because the general operational processes (e.g., Darwinian evolution, mutualism, parasitism, etc.) do not differ, studies utilizing digital networks can uncover rules operating on and within ecological networks. Together with microbial experiments, they create opportunities for furthering the understanding of the interplay between ecological and evolutionary processes among interacting species.

## Supporting Information

Text S1 Version history of the text file.
(XML)

Text S2 Peer reviews and response to reviews. Human-readable versions of the reviews and authors' responses are available as comments on this article.
(XML)

Video S1 Self-replication of a digital organism. The circular genome of a digital organism, on the left, consists of a set of instructions (represented here as letters). Some of these instructions are involved in the copy process and others in completing computational tasks. The experimenter determines the probability of mutations. Copy mutations occur when an instruction is copied incorrectly, and is instead replaced by a random instruction in the forming offspring's genome (as can be seen in the offspring, on the right). Other types of mutations, such as insertions and deletions, are also implemented. Initially, all three of the parent's hardware pointers are in the same location, at the instruction represented here by *r*. As execution begins, the instruction pointer (indicated by an *i*) advances. The first few instructions allocate space for the offspring, and then move the write-head pointer (indicated by a *w*) into that space. The flow pointer (indicated by an *f*) is used to move the other pointers to genetically specified locations. The remainder of the process of self replication is carried out by a set of instructions at the end of the genome, commonly referred to as the copy-loop. When execution reaches the copy-loop, the flow pointer is used to keep the flow of execution inside of a loop that advances the read and write heads and copies instructions from the parent genome (read-head) to the offspring genome (write-head). Arcs inside the circular genome represent the execution flow, showing most of the CPU cycles being used during the copying process. After genome replication is complete, the parent organism divides off its offspring, which must now fend for itself within the Avida world. This animation was generated using Avida-ED, which is available under the terms of the GNU Lesser General Public License at http://avida-ed.msu.edu/.
(OGV)

Video S2 Evolving host–parasite webs. Starting from a host phenotype (green node) and a parasite phenotype (red node), a complex network of interactions (arrows) between hosts and parasites emerges out of the coevolutionary process. Nodes representing new host and parasite phenotypes appear and disappear over evolutionary time. The abundance of individuals expressing each phenotype changes continuously (indicated by node size) altering interaction patterns, and thus influencing subsequent coevolutionary dynamics. Interactions between a host phenotype and a parasite phenotype are depicted as arrows pointing in opposite directions: the thickness of red arrows indicates the fraction of infections that a particular parasite is responsible for inflicting on the indicated host phenotype, while the thickness of the green arrows indicates the fraction of all of the hosts a particular parasite phenotype infects that is accounted for by the indicated host phenotype. Often asymmetry between the thicknesses of arrow-pairs leads to red arrows dominating the picture. At these times, most parasite phenotypes are infecting only a small fraction of hosts expressing a given phenotype. Instead, the majority of those hosts are being infected by parasites with other phenotypes. This animation was generated using Pajek, which is available under the GNU General Public License at http://pajek.imfm.si/doku.php.
(OGV)
